# Letter to the Editor: Cautionary Note on Ribonuclease Activity of Recombinant PR-10 Proteins

**DOI:** 10.1093/pcp/pcad062

**Published:** 2023-06-15

**Authors:** Rawit Longsaward, Nattapong Sanguankiattichai, Unchera Viboonjun, Renier A L van der Hoorn

**Affiliations:** The Plant Chemetics Laboratory, Department of Biology, University of Oxford, Oxford OX1 3RB, UK; Department of Plant Science, Faculty of Science, Mahidol University, Bangkok 10400, Thailand; Department of Plant Pathology, Faculty of Agriculture, Kasetsart University, Bangkok 10900, Thailand; The Plant Chemetics Laboratory, Department of Biology, University of Oxford, Oxford OX1 3RB, UK; Department of Plant Science, Faculty of Science, Mahidol University, Bangkok 10400, Thailand; The Plant Chemetics Laboratory, Department of Biology, University of Oxford, Oxford OX1 3RB, UK

Class 10 of pathogenesis-related proteins (PR-10) is a class of PR proteins localized in the cytoplasm (Agarwal and Agarwal 2014). Members of the PR-10 protein family are part of the major latex-like protein family and can be induced by the infection with various pathogens (Fujita and Inui 2021). Recently, we identified a novel PR-10 protein from rubber tree (*Hevea brasiliensis*), previously known as protein LOC110648447, which accumulates in leaves in response to root infection by white root rot fungi, *Rigidoporus microporus* (Longsaward et al. 2023). Sequence similarity and predicted 3D structure indicate that *Hb*PR10 is a member of the Bet v 1/PR-10 protein family.

The *HbPR10* gene consists of four exons that can be combined in three ways (**Fig. 1A**), encoding *Hb*PR10.1, *Hb*PR10.2 and *Hb*PR10.3, which differ from each other at several residues (**Supplementary Fig. S1**). To investigate *Hb*PR10s, we cloned the three splicing isoforms into pET28b with a C-terminal His-tag (hexahistidine, **Supplementary Table S1**). The plasmids were confirmed by sequencing and transformed into *Escherichia coli* Rosetta DE3 (Sigma Aldrich, St. Louis, MO, USA, **Supplementary Table S2**). Protein expression was induced with 0.2 mM isopropylthio-β-galactoside (IPTG), cultures were lysed with CellLytic Express buffer (Sigma Aldrich) and His-tagged proteins were purified on Ni-NTA agarose resin (ThermoFisher, Loughborough, Leicestershire, UK). The column was extensively washed and eluted with PBS buffer containing 50 and 250 mM imidazole, respectively. All three *Hb*PR10 proteins are soluble and produced and purified with high yields (**Fig. 1B** and **Supplementary Fig. S2**).

**Fig. 1:**
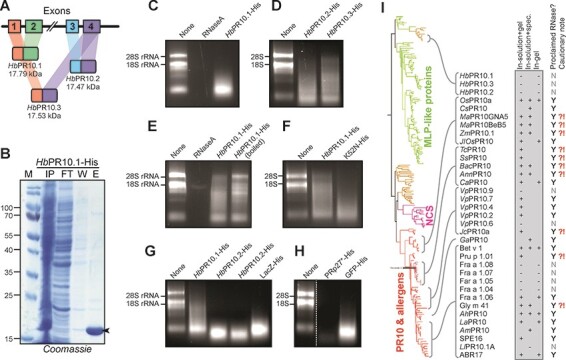
Proclaimed ribonuclease activity of PR-10 proteins should be interpreted with caution. (A) The alternative splicing among three *Hb*PR10 isoforms according to the reference sequence in the National Center for Biotechnology Information database (NW_01874736.1). The calculated molecular weights are shown. (B) The expression and purification of *Hb*PR10.1-His from *E. coli*. Lanes IP, FT, W and E show the protein profile from each of the Ni-NTA resin purification steps including the input, flow-through, wash and eluate, respectively. (C) In-solution ribonuclease activity of *Hb*PR10.1. (D) In-solution ribonuclease activity of *Hb*PR10.2 and *Hb*PR10.3. (E) In-solution ribonuclease activity is reduced upon boiling *Hb*PR10.1. (F) In-solution ribonuclease activity is unaltered in the K52N mutant of *Hb*PR10.1. (G) In-solution ribonuclease activity by purified *Hb*PR10s and LacZ. (H) In-solution ribonuclease activity by purified PRp27 and GFP. (C–H) The total plant RNA (1.25 μg) was incubated with PBS buffer or purified (boiled/mutant) proteins (1 μg) at 37^o^C for 180 min in a total reaction volume of 12 μl, followed by 1% agarose gel electrophoresis. (I) PR-10 homologs studied in ribonuclease assays are ranked phylogenetically. Ribonuclease assays are as follows: (i) in-solution ribonuclease assay, followed by agarose gel electrophoresis; (ii) in-solution ribonuclease assay, followed by spectrometric analysis and (iii) in-gel ribonuclease assay. Ribonuclease activity was observed (+) or not (−), resulting in proclaimed ribonuclease activities (Y). Proteins with proclaimed ribonuclease activity but insufficient controls are noted with a bold question/caution mark (**?!**). The maximum-likelihood tree was generated with the PhyML server.

A frequently reported characteristic of PR-10 proteins is the ability to cleave RNA in solution, detected by the separation of RNA on agarose gels (Liu et al. 2006, Kim et al. 2008, Pungartnik et al. 2009, Zubini et al. 2009, Agarwal et al. 2013). Ribonuclease activity of PR-10 is thought to have a direct effect on viral RNA or on general RNA homeostasis during defense. We used this in-solution ribonuclease assay to test if *Hb*PR10s have ribonuclease activity. We incubated 1.25 μg total RNA, which was extracted from *Nicotiana benthamiana* leaves via Trizol-based RNA extraction (ThermoFisher) with 1 μg of purified *Hb*PR10.1-His protein for 180 min at 37^o^C, separated the RNA on 1% agarose gel and stained the gel with ethidium bromide. In the presence of *Hb*PR10.1-His, the ribosomal RNA (rRNA) was degraded and low-molecular-weight signals appeared (**Fig. 1C**). The RNA was completely degraded by RNaseA (positive control) (**Fig. 1C**). Similar ribonuclease activities were observed with purified *Hb*PR10.2-His and *Hb*PR10.3-His (**Fig. 1D**).

Heat inactivation is an often-used negative control in ribonuclease studies (Liu et al. 2006, Pungartnik et al. 2009, Zubini et al. 2009, Xie et al. 2010, Agarwal et al. 2013, Fan et al. 2015). Heat treatment of *Hb*PR10-His (95^o^C for 15 min), followed by incubation with RNA, resulted in a reduced RNA degradation compared to unheated *Hb*PR10.1-His (**Fig. 1E**). We next generated the K52N substitution mutant of *Hb*PR10.1 because this mutation was found to abolish RNase activity in *Ah*PR10 (Chadha and Das 2006). However, *Hb*PR10.1^K52N^-His is still able to degrade RNA in our in-solution assays (**Fig. 1F**).

Other studies on PR-10 proteins included a protein with no reported ribonuclease activity as a negative control for the ribonuclease assay. For instance, bovine serum albumin (BSA) was included as a negative control for the in-solution ribonuclease assay of rice *Os*PR10 (Kim et al. 2008) and SPE16, a PR-10 protein from jicama Mexican turnip (Wu et al. 2002). Likewise, a GST-tagged protein was included as a control for grape *Vp*PR10s (He et al. 2013, Wang et al. 2014). These negative controls, however, were not produced and purified in the same way as for the PR-10 protein. In order to confirm whether the detected ribonuclease activity was specifically from *Hb*PR10, we included a β-galactosidase with a C-terminal His-tag encoded by *LacZ*, which can cleave lactose but not RNA, as a negative control. LacZ-His was cloned into the same plasmid backbone and purified using the same methods as for the three *Hb*PR10 proteins (**Supplementary Fig. S2**). Alarmingly, however, purified LacZ-His also caused RNA degradation, similar to all three *Hb*PR10s (**Fig. 1G**). Likewise, GFP-His and an inactive mutant of PRp27 (PRp27^H122F^-His, Morimoto et al. 2022), expressed in *E. coli* and purified following the same procedure, were also able to degrade RNA (**Fig. 1H**). These experiments indicate that the ribonuclease activity may originate from some copurified proteins present in all samples.

Based on the results we obtained here, *Hb*PR10 proteins are unproven to have ribonuclease activity at the conditions tested because we detected ribonuclease activity also for the negative control proteins. We found that the nickel affinity column for purification cannot eliminate copurified contaminant ribonucleases and hence cause RNA degradation, even by negative control proteins. Even though the in-solution assay is easy and frequently used (**Fig. 1I**, **Supplementary Table S3**), copurified ribonucleases contaminating the eluates are easily overlooked. Inactivation by boiling or inactivation by using ribonuclease inhibitors is insufficient as a negative control, and also the use of proteins that were not produced and purified following the same procedure are unsuitable controls. Consequently, previous conclusions that *Ss*PR10 (Liu et al. 2006), Pru p s (Zubini et al. 2009), *Tc*PR-10 (Pungartnik et al. 2009), *Zm*PR10.1 (Xie et al. 2010), *ann*PR10 and *bac*PR10 (Soh et al. 2012), *Jc*PR-10a  (Agarwal et al. 2013), Gly m 41 (Fan et al. 2015), *Pn*PR-like (Li et al. 2021) and *Ma*PR10s (Rajendram et al. 2022) have ribonuclease activities should be interpreted with caution, given the absence of proper controls. In-gel ribonuclease assays offer more robust detection of ribonuclease activity although copurified RNases might also cause gel clearings. The origin of the gel clearing can be linked to the PR-10 when inactive mutants are included or when the gel clearing shifts upward in the gel when the PR-10 protein is fused with another protein.

The absence of detectable ribonuclease activity for *Hb*PR10 is consistent with other reported PR-10 proteins that lack measurable ribonuclease activity such as *Vp*PR10.6 and *Vp*PR10.9 (Wang et al. 2014). The construction of an evolutionary tree with reported PR-10 protein homologs (**Fig. 1I**) showed that prematurely proclaimed ribonuclease activities are common across the PR-10 subfamily. *Hb*PR10 proteins are members of the major latex protein (MLP) subgroup of the PR-10 family. A review article by Fujita and Inui (2021) suggested that no MLP proteins were reported with ribonuclease activity despite being tested for this activity. Therefore, our experiment attempted to be the first report of ribonuclease activity from the MLP-related subgroup of the PR-10 protein (**Fig. 1I**). We hope that this cautionary note will encourage the community to develop appropriate assays and include adequate negative controls to detect ribonuclease activity. Given the sensitivity for RNA degradation by contaminant RNAses, mutant PR-10 proteins that lack ribonuclease activity but are produced and purified in the same way are essential negative controls for future assays.

## Supplementary Material

pcad062_Supp

## Data Availability

All data generated or analyzed during this study are included in this published article and its supplementary information file.

## References

[R1] Agarwal P. and Agarwal P.K. (2014) Pathogenesis related-10 proteins are small, structurally similar but with diverse role in stress signaling. *Mol. Biol. Rep.* 41: 599–611. 24343423 10.1007/s11033-013-2897-4

[R2] Agarwal P. , Bhatt V., Singh R., Das M., Sopory S.K. and Chikara J. (2013) Pathogenesis-related gene, JcPR-10a from *Jatropha curcas* exhibit RNase and antifungal activity. *Mol. Biotechnol.* 54: 412–425. 22763562 10.1007/s12033-012-9579-7

[R3] Chadha P. and Das R.H. (2006) A pathogenesis related protein, AhPR10 from peanut: an insight of its mode of antifungal activity. *Planta* 225: 213–222. 16832688 10.1007/s00425-006-0344-7

[R4] Fan S.J. , Jiang L.Y., Wu J.J., Dong L.D., Cheng Q., Xu P.F., et al. (2015) A novel pathogenesis-related class 10 protein *Gly m 41*, increases resistance upon *Phytophthora sojae* infection in soybean (*Glycine max* [L.] Merr.). *PLoS One* 10: e0140364. 10.1371/journal.pone.0140364 PMC4608668 26474489

[R5] Fujita K. and Inui H. (2021) Review: biological functions of major latex-like proteins in plants. *Plant Sci.* 306: 110856. 10.1016/j.plantsci.2021.110856 33775363

[R6] He M.Y. , Xu Y., Cao J.L., Zhu Z.G., Jiao Y.T., Wang Y.J., et al. (2013) Subcellular localization and functional analyses of a PR10 protein gene from *Vitis pseudoreticulata* in response to *Plasmopara viticola* infection. *Protoplasma* 250: 129–140. 22327469 10.1007/s00709-012-0384-8

[R7] Kim S.T. , Yu S., Kang Y.H., Kim S.G., Kim J.Y., Kim S.H., et al. (2008) The rice pathogen- related protein 10 (JIOsPR10) is induced by abiotic and biotic stresses and exhibits ribonuclease activity. *Plant Cell Rep.* 27: 593–603. 18074138 10.1007/s00299-007-0485-6

[R8] Liu X. , Huang B., Lin J., Fei J., Chen Z., Pang Y., et al. (2006) A novel pathogenesis- related protein (SsPR10) from *Solanum surattense* with ribonucleolytic and antimicrobial activity is stress- and pathogen-inducible. *J. Plant Physiol.* 163: 546–556. 16473659 10.1016/j.jplph.2005.04.031

[R9] Li S. , Wang Z., Tang B., Zheng L., Chen H., Cui X., et al. (2021) A pathogenesis-related protein-like gene is involved in the *Panax notoginseng* defense response to the root rot pathogen. *Front. Plant Sci.* 11: 610176. 10.3389/fpls.2020.610176 PMC7838351 33519865

[R10] Longsaward R. , Pengnoo A., Kongsawadworakul P. and Viboonjun U. (2023) A novel rubber tree PR-10 protein involved in host-defense response against the white root rot fungus *Rigidoporus microporus*. *BMC Plant Biol.* 23: 157. 10.1186/s12870-023-04149-3 PMC10032002 36944945

[R11] Morimoto K. , Krahn D., Kaschani F., Hopkinson-Woolley D., Gee A., Buscaill P., et al. (2022) Broad-range metalloprotease profiling in plants uncovers immunity provided by defence-related metalloenzyme. *New Phytol.* 235: 1287–1301. 35510806 10.1111/nph.18200 PMC9322406

[R12] Pungartnik C. , Clara da Silva A., Alves de Melo S., Gramacho K.P., Cascardo J.C.M., Brendel M., et al. (2009) High-affinity copper transport and Snq2 export permease of *Saccharomycetes cerevisiae* modulate cytotoxicity of PR-10 from *Theobroma cacao*. *Mol. Plant Microbe Interact.* 22: 39–51. 19061401 10.1094/MPMI-22-1-0039

[R13] Rajendram A. , Mostaffa N.H., Dumin W., Oke M.A., Simarani K., Somasundram C., et al. (2022) Dual activity of *Meloidogyne incognita*-regulated *Musa acuminata Pathogenesis-related-10* (*MaPR-10*) gene. *Gene* 809: 146041. 10.1016/j.gene.2021.146041 34710526

[R14] Soh H.C. , Park A.R., Park S., Back K., Yoon J.B., Park H.G., et al. (2012) Comparative analysis of pathogenesis-related protein 10 (*PR10*) genes between fungal resistant and susceptible peppers. *Eur. J. Plant Pathol.* 132: 37–48.

[R15] Wang L. , Wei J., Zou Y., Xu K., Wang Y., Cui L., et al. (2014) Molecular characteristics and biochemical functions of VpPR10s from *Vitis pseudoreticulata* associated with biotic and abiotic stresses. *Int. J. Mol. Sci.* 15: 19162–19182. 25340981 10.3390/ijms151019162 PMC4227267

[R16] Wu F. , Li Y., Chang S., Zhou Z., Wang F., Song X., et al. (2002) Purification, characterization and preliminary crystallographic studies of a PR-10 protein from *Pachyrrhizus erosus*seeds. *Acta Crystallogr. D Biol. Crystallogr.* 58: 2165–2167. 12454488 10.1107/s0907444902016062

[R17] Xie Y.R. , Chen Z.Y., Brown R.L. and Bhatnagar D. (2010) Expression and functional characterization of two pathogenesis-related protein 10 genes from *Zea mays*. *J. Plant Physiol.* 167: 121–130. 19682768 10.1016/j.jplph.2009.07.004

[R18] Zubini P. , Zambelli B., Musiani F., Ciurli S., Bertolini P. and Baraldi E. (2009) The RNA hydrolysis and the cytokinin binding activities of PR-10 proteins are differently performed by two isoforms of the Pru p 1 peach major allergen and are possibly functionally related. *Plant Physiol.* 150: 1235–1247. 19474212 10.1104/pp.109.139543 PMC2705045

