# Supplementary material for: Letter to the Editor: Cautionary Note on Ribonuclease Activity of Recombinant PR-10 Proteins
Source: Plant Cell Physiol. 2023 Jun 15;64(8):847–9. doi: 10.1093/pcp/pcad062 (PMC10434734; doi:10.1093/pcp/pcad062)
Supplement: pcad062_Supp [file pcad062_supp.zip › suppl_data/pcp-2023-e-00087-File003.pdf]

Supplemental Tables S1-S2-S3

Cautionary note on ribonuclease activity of recombinant PR-10 proteins

Rawit Longsaward, Nattapong Sanguankiattichai, Unchera Viboonjun, Renier A.L. van der Hoorn

Table S1 Oligonucleotides used in this study

Fragment	Primer name	Sequence (5' to 3')
*Hb*PR10.1-His	PR10_pET28b_F	CCCTCTAGAAATAATTTTGTTTAACTTTAAGAAGGAGATATACAATGGCTTTCGTGACTGCTACTGCT
	PR10_pET28b_R1	GTTAGCAGCCGGATCCAAGCCTAGTGATGGTGATGGTGATGGGTCAGCTTGTTCTGGATGTAG
*Hb*PR10.2-His	PR10_pET28b_F	CCCTCTAGAAATAATTTTGTTTAACTTTAAGAAGGAGATATACAATGGCTTTCGTGACTGCTACTGCT
	PR10_pET28b_R2	GTTAGCAGCCGGATCCAAGCCTAGTGATGGTGATGGTGATGGGTGCTCTTGTTCTGGATGTAG
*Hb*PR10.3-His	PR10_pET28b_F	CCCTCTAGAAATAATTTTGTTTAACTTTAAGAAGGAGATATACAATGGCTTTCGTGACTGCTACTGCT
	PR10_pET28b_R2	GTTAGCAGCCGGATCCAAGCCTAGTGATGGTGATGGTGATGGGTGCTCTTGTTCTGGATGTAG
His-GFP-Strep	oNS368	CCCTCTAGAAATAATTTTGTTTAACTTTAAGAAGGAGATATACAATGGCGCATCACCATCACCATC
	oNS369	GTTAGCAGCCGGATCCAAGCTCACTTTTCGAACTGCGGGTGGCTCCAGCTACCTTTGTACAGTTCATCCATACCATGCG
LacZ-His	oNS339	CCCTCTAGAAATAATTTTGTTTAACTTTAAGAAGGAGATATACAATGACCATGATTACGGATTCAC
	oNS340	GTTAGCAGCCGGATCCAAGCTTAGTGATGGTGATGGTGATGTTTTTGACACCAGACCAACT

Table S2 Plasmids used in this study

Plasmid	Description	Reference
pRL010	*Hb*PR10.1-His in pET28b vector	*This work*
pRL011	*Hb*PR10.2-His in pET28b vector	*This work*
pRL012	*Hb*PR10.3-His in pET28b vector	*This work*
pNS141	LacZ-His in pET28b vector	*This work*
pNS153	His-GFP-Strep in pET28b vector	*This work*
pKM008	His-PRp27(H122F)	Morimoto et al., 2022

Supplemental **Table S3** Ribonuclease assays on PR-10 protein used in previous studies.

Types	Protein	Accession no.	Original species	Heterologous expression in *E. coli.*	Fusion tag	Purification method	Downstream process			RNase assay			Negative controls							Reference
							Dialyzed	Fractioned	Remove tag	In-solution and agarose gel electrophoresis	In-solution and spectrophotometry	In-gel ribonuclease	Boiled protein	Mutant protein	RNase inhibitor	Buffer (no protein)	(+) DTT	Empty vector	Other protein	
	*Ca*PR10	AAF63519.1	*Capsicum annuum*	-	-	Crude protein extraction	-	-	-	+	-	+	-	-	+	+	+	+	-	Park et al. 2004
Native	SPE16	ARR11455.1	*Pachyrrhizus erosus*	-	-	DEAE-Sepharose column	-	+	-	+	-	-	-	-	-	+	-	-	+	Wu et al. 2002
Native	*Os*PR10a	XP_015620382.1	*Oryza sativa*	-	-	Continuous-elution electrophoresis	-	+	-	+	+	+	-	-	-	+	-	-	-	Huang et al. 2016
Native	Bet v 1	1FM4_A	*Betula alba*	-	-	Fast protein liquid chromatography (FPLC)	+	+	-	+	+	+	-	-	+	+	-	-	-	Bufe et al. 1996
Native	*Am*PR10	ASA69247.1	*Astragalus mongholicus*	-	-	Phosphate buffer	+	+	-	-	+	-	-	-	-	-	-	-	-	Yan et al. 2008
Native	Bet v 1	1FM4_A	*Betula alba*	-	-	Reverse phase HPLC	-	-	-	+	-	+	-	-	-	+	-	-	-	Swoboda et al. 1996
Native	*As*PRs	-	*Angelica sinensis*	-	-	Sephadex G50; Ion exchange				-	+	-	-	-	-	-	-	-	-	Pan et al. 2018
Recombinant	Fra a 1s	AHZ10955.1, AHZ10956.1, AHZ10957.1, AHZ10958.1, AHZ10959.1	*Fragaria x ananassa*	+	C-term hexahistidine	Affinity purified	-	-	-	+	-	+	+	-	-	+	-	+	-	Besbes et al. 2019

Recombinant	*Cs*PR10	ADL09408.1	*Crocus sativus*	+	GST tag	GS-4B resin, followed by MALDI analysis	-	-	-	-	+	-	-	-	-	-	+	-	-	Gomez-Gomez et al. 2011
Recombinant	*Ann*PR10	ABC74798.1	*Capsicum annuum*	+	GST tag	GST tag purification	-	-	-	+	+	-	-	-	-	+	-	-	-	Soh et al. 2012
Recombinant	*Bac*PR10	ABC74797.1	*Capsicum baccatum*	+	GST tag	GST tag purification	-	-	-	+	+	-	-	-	-	+	-	-	-	Soh et al. 2012
Recombinant	*Vp*PR10.2	ABD78556.1	*Vitis pseudoreticulata*	+	GST tag	GST tag purification	-	-	-	+	-	-	+	-	+	-	-	-	+	He et al. 2013
Recombinant	*Vp*PR10s	-	*Vitis pseudoreticulata*	+	GST tag	GST tag purification	-	-	-	+	-	-	+	-	-	+	-	-	+	Wang et al. 2014
Recombinant	Gly m 41	ADX43926.1	*Glycine max*	+	Hexahistidine	His-bind resin column	-	-	-	+	+	-	+	-	-	+	-	-	-	Fan et al. 2015
Recombinant	*Ss*PR10	AAU00066.1	*Solanum surattense*	+	Polyhistidine	His-bond Ni Affinity resin column	+	-	-	+	-	-	+	-	-	+	-	+	-	Liu et al. 2006
Recombinant	SPE16	ARR11455.1	*Pachyrrhizus erosus*	+	n/a	Ni-chelating Sepharose Fast Flow Gel column	-	-	-	+	-	-	-	+	-	+	-	-	-	Wu e al. 2003
Recombinant	*Zm*PR10.1	ADA68331.1	*Zea mays*	+	C-term hexahistidine	Ni-IDA affinity column	-	-	-	+	+	-	+	-	+	-	-	+	-	Xie et al. 2010
Recombinant	ABR17 (PR10.4)	Q06931.1	*Pisum saivum*	+	N-term hexahistidine	Ni-NTA agarose column	+	-	+	+	-	+	+	-	-	-	-	-	-	Srivastava et al. 2006a, 2007
Recombinant	ABR17 (PR10.4)	Q06931.1	*Pisum saivum*	+	N-term hexahistidine	Ni-NTA agarose column	+	-	-	+	-	-	+	+	-	-	-	-	-	Krishnaswamy et al. 2011
Recombinant	*Ca*PR10	AAF63519.1	*Capsicum annuum*	+	N-term hexahistidine	Ni-NTA agarose column	-	-	-	+	-	-	+	-	-	+	-	+	-	Park et al. 2004
Recombinant	*Ga*PR-10	AAL09033.1	*Gossypium arboreum*	+	N-term hexahistidine	Ni-NTA agarose column	-	-	+	-	+	-	-	-	-	-	-	-	-	Zhou et al. 2002
Recombinant	*Jc*PR-10a	AEV54115.1	*Jatropa curcas*	+	Hexahistidine	Ni-NTA agarose column	-	-	-	+	-	-	+	-	-	+	-	-	-	Agarwal et al. 2012
Recombinant	*JIOs*PR10	AAL74406.1	*Oryza sativa*	+	C-term hexahistidine	Ni-NTA agarose column	-	-	-	+	-	+	-	+	+	+	+	-	+	Kim et al. 2008
recombinant	*La*PR-10	CAA03926.1	*Lupinus albus*	+	C-term hexahistidine	Ni-NTA agarose column	-	-	-	+	-	+	-	-	+	-	-	-	-	Bantignies et al. 2000
Recombinant	Pea PR10.1	-	*Pisum saivum*	+	N-term hexahistidine	Ni-NTA agarose column	+	-	+	+	-	+	+	-	-	+	-	-	-	Srivastava et al. 2006b

Recombinant	*Pn*PR-like	QOJ53932.1	*Panax notoginseng*	+	Polyhistidine	Ni-NTA agarose column	-	-	-	+	-	-	-	-	+	+	-	-	-	Li et al. 2021
Recombinant	Pru p 1s	ACE80940.1	*Prunus persica*	+	N-term hexahistidine	Ni-NTA agarose column	+	-	-	+	-	-	+	-	-	+	-	+	-	Zubini et al. 2009
Recombinant	*Ma*PR10s	UED15064.1, UED15065.1	*Musa acuminata*	+	N-term hexahistidine	Nickle His Gravitrap affinity column	-	-	-	+	+	-	-	-	-	+	-	-	-	Rajendram et al. 2022
Recombinant	Bet v 1	1FM4_A	*Betula alba*	+	-	PBE-94 exchange column	-	+	-	-	-	+	-	-	-	+	-	-	-	Swoboda et al. 1996
Recombinant	*Ah*PR10	AAU81922.1	*Arachis hypogaea*	+	C-term hexahistidine	Talon resin metal affinity column	-	+	-	+	+	+	-	+	-	+	-	-	-	Chadha and Das 2006
Recombinant	*Tc*PR-10	-	*Theobroma cacao*	+	N-term hexahistidine	Talon resin metal affinity column	+	-	-	+	-	-	+	-	-	+	-	-	-	Pungartnik et al. 2009

Note: "+" indicates the method used in the study and "-" indicates the method was not used in the study.

REFERENCES

Agarwal, P., Bhatt, V., Singh, R., Das, M., Sopory, S. K., and Chikara, J. (2012) Pathogenesis-related gene, *Jc*PR-10a from *Jatropha curcas* exhibit RNase and antifungal activity. *Mol. Biotechn.* **54**, 412-425.

Bantignies B., Seguin J., Muzac I., Dedaldechamp F., Gulick P., and Ibrahim R. (2000) Direct evidence for ribonucleolytic activity of a PR-10-like protein from white lupin roots. *Plant Mol. Biol.* **42**, 871-881.

Besbes, F., Habegger, R., and Schwab, W. (2019) Induction of PR-10 genes and metabolites in strawberry plants in response to *Verticillium dahlia* infection. *BMC Plant Biol.* **19**, 128.

Bufe A., Spangfort M. D., Kahlert H., Schlaak M., and Becker W. M. (1996) The major birch pollen allergen, Bet v 1, shows ribonuclease activity. *Planta* **199**, 413-415.

Chadha P., and Das R. H. (2006) A pathogenesis related protein, *Ah*PR10 from peanut: an insight of its mode of antifungal activity. *Planta* **225**, 213-222.

Fan, S. J., Jiang, L. Y., Wu, J. J., Dong, L. D., Cheng, Q., Xu, P. F., and Zhang, S. Z. (2015) A novel pathogenesis-related class 10 protein Gly m 41, increases resistance upon *Phytophthora sojae* infection in soybean (Glycine max [L.] Merr.). *PLoS ONE* **10**, e0140364.

Gomez-Gomez, L., Rubio-Moraga, A., and Ahrazem, O. (2011) Molecular cloning and characterization of a pathogenesis-related protein *Cs*PR10 from *Crocus sativus*. *Plant Biol.* **13**, 297-303.

He, M. Y., Xu, Y., Cao, J. L., Zhu, Z. G., Jiao, Y. T., Wang, Y. J., Xin, G., Yang, Y. Z., Xu, W. R., and Fu, Z. F. (2013) Subcellular localization and functional analyses of a PR10 protein gene from *Vitis pseudoreticulata* in response to *Plasmopara viticola* infection. *Protoplasma* **250**, 129-140.

Huang, L. F., Lin, K. H., He, S. L., Chen, J. L., Jiang, J. Z., Chen, B. H., Hou, Y. S., Chen, R. S., Hong, C. Y., and Ho, S. L. (2016) Multiple patterns of regulation and overexpression of a ribonuclease-like pathogenesis-related protein gene, *Os*PR10a, conferring disease resistance in rice and Arabidopsis. *PLoS ONE* **11**: e0156414.
Kim, S. T., Yu, S., Kang, Y. H., Kim, S. G., Kim, J. Y., Kim, S. H., and Kang, K. Y. (2008) The rice pathogen- related protein 10 (JIOsPR10) is induced by abiotic and biotic stresses and exhibits ribonuclease activity. *Plant Cell Rep.* **27**, 593-603.
Krishnaswamy, S., Baral, P. K., James, M. N. G., and Kav, N. N. V. (2011) Site-directed mutagenesis of histidine 69 and glutamic acid 148 alters the ribonuclease activity of pea ABR17 (PR10.4). *Plant Physiol. Biochem.* **49**, 958-962.
Li, S., Wang, Z., Tang, B., Zheng, L., Chen, H., Cui, X., Ge, F., and Liu, D. (2021) A pathogenesis-related protein-like gene is involved in the *Panax notoginseng* defense response to the root rot pathogen. *Front. Plant Sci.* **11**, 610176.
Liu, X., Huang, B., Lin, J., Fei, J., Chen, Z., Pang, Y., Sun, X., and Tang, K. (2006) A novel pathogenesis- related protein (*Ss*PR10) from *Solanum surattense* with ribonucleolytic and antimicrobial activity is stress- and pathogen-inducible. *J. Plant Physiol.* **163**, 546-556.
Pan, J., Wang, X., Li, L., Li, X., Ye, X., Lv, D., Chen, C., Liu, S., and He, H. (2018) Purification and characterization of two pathogenesis-related class 10 protein isoforms with ribonuclease activity from the fresh *Angelica sinensis* roots. *Plant Physiol. Biochem.* **128**, 66-71.
Park, C. J., Kim, K. J., Shin, R., Park, J. M., Shin, Y. C., and Paek, K. H. (2004) pathogenesis-related protein 10 isolated from hot pepper functions as a ribonuclease in an antiviral pathway. *Plant J.* **37**, 186-198.
Pungartnik, C., Clara da Silva, A., Alves de Melo, S., Gramacho, K. P., Cascardo, J. C. M., Brendel, M., Micheli, F., and Gesteira, A. S. (2009) High-affinity copper transport and Snq2 export permease of *Saccharomycetes cerevisiae* modulate cytotoxicity of PR-10 from *Theobroma cacao*. *Mol. Plant-microbe Interact.* **22**, 39-51.
Rajendram, A., Mostaffa, N. H., Dumin, W., Oke, M. A., Simarani, K., Somasundram, C., Razali, Z., Rejab, N. A., and Al-Idrus, A. (2022) Dual activity of Meloidogyne incognita-regulated *Musa acuminata* Pathogenesis-related-10 (*Ma*PR-10) gene. *Gene* **809**, 146041.
Soh, H. C., Park, A. R., Park, S., Back, K., Yoon, J. B., Park, H. G., and Kim, Y. S. (2012) Comparative analysis of pathogenesis-related protein 10 (PR10) genes between fungal resistant and susceptible peppers. *Eur. J. Plant Pathol.* **132**, 37-48.
Srivastava, S., Rahman, M. H., Shah, S., and Kav, N. N. V. (2006a) Constitutive expression of the pea ABA-responsive (ABR17) cDNA confers multiple stress tolerance in *Arabidopsis thaliana*. *Plant Biotechnol. J.* **4**, 529-549.
Srivastava, S., Emery, R. J. N., Kurepin, L. V., Reid, D. M., Fristensky, B., and Kav, N. N. V. (2006b) Pea PR 10.1 is a ribonuclease and its transgenic expression elevates cytokinin levels. *Plant Growth Regul.* **49**, 17-25.
Srivastava, S., Emery, R. J. N., Rahman, M. H., and Kav, N. N. V. (2007) A crucial role for cytokinins in pea ABR17-mediated enhanced germination and early seedling growth of *Arabidopsis thaliana* under saline and low-temperature stresses. *J. Plant Growth Regul.* **26**, 26-37.
Swoboda, I., Sommergruber, K. H., O'Riordain, G., Scheiner, O., Bors, E. H., and Vicente, O. (1996) Bet v 1 proteins, the major birch pollen allergens and members of a family of conserved pathogenesis-related proteins, show ribonuclease activity *in vitro*. *Physiologia Plantarum* **96**, 433-438.
Wang, L., Wei, J., Zou, Y., Xu, K., Wang, Y., Cui, L., and Xu, Y. (2014) Molecular characteristics and biochemical functions of *Vp*PR10s from *Vitis pseudoreticulata* associated with biotic and abiotic stresses. *Int. J. Mol. Sci.* **15**, 19162-19182.

Wu, F., Li, Y., Chang, S., Zhou, Z., Wang, F., Song, X., Lin, Y., and Gong, W. (2002) Purification, characterization and preliminary crystallographic studies of a PR-10 protein from *Pachyrrhizus erosus* seeds. *Acta Crystallogr D Biol Crystallogr* **58**, 2165-2167.
Wu, F., Yan, M., Li, Y., Chang, S., Song, X., Zhou, Z., and Gong, W. (2003) cDNA cloning, expression, and mutagenesis of a PR-10 protein SPE-16 from the seeds of *Pachyrrhizus erosus*. *Biochem. Biophys. Res. Comm.* **312**, 761-766.
Xie, Y. R., Chen, Z. Y., Brown, R. L., and Bhatnagar, D. (2010) Expression and functional characterization of two pathogenesis-related protein 10 genes from *Zea mays*. *J. Plant Physiol.* **167**, 121-130.
Yan Q. J., Qi X. W., Jiang Z. Q., Yang S. Q., and Han L. J. (2008) Characterization of a pathogenesis- related class 10 protein (PR-10) from *Astragalus mongholicus* with ribonuclease activity. *Plant Physiol. Biochem.* **46**, 93-99.
Zhou X. J., Lu S., Xu Y. H., Wang J. W., and Chen X. Y. (2002) A cotton cDNA (GaPR-10) encoding a pathogenesis-related 10 protein with *in vitro* ribonuclease activity. *Plant Sci.* **162**, 629-636.
Zubini, P., Zambelli, B., Musiani, F., Ciurli, S., Bertolini, P., and Baraldi, E. (2009) The RNA hydrolysis and the cytokinin binding activities of PR-10 proteins are differently performed by two isoforms of the Pru p 1 peach major allergen and are possibly functionally related. *Plant Physiol.* **150**, 1235-1247.
