# Supplementary material for: Letter to the Editor: Cautionary Note on Ribonuclease Activity of Recombinant PR-10 Proteins
Source: Plant Cell Physiol. 2023 Jun 15;64(8):847–9. doi: 10.1093/pcp/pcad062 (PMC10434734; doi:10.1093/pcp/pcad062)
Supplement: pcad062_Supp [file pcad062_supp.zip › suppl_data/pcp-2023-e-00087-File004.pdf]

Supplemental Figures

Cautionary note on ribonuclease activity of recombinant PR-10 proteins

Rawit Longsaward[1,2], Nattapong Sanguankiattichai[1], Unchera Viboonjun[2], Renier A.L. van der Hoorn[1,*]

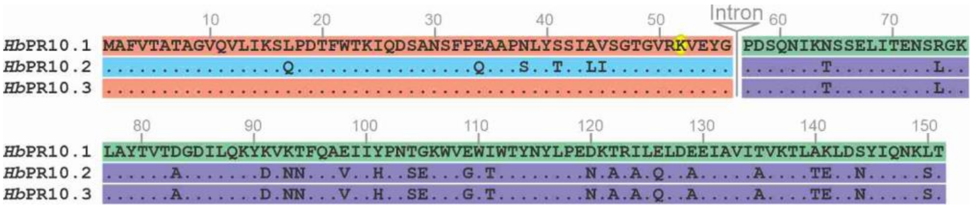

Supplemental Figure S1 Protein alignment of *Hb*PR10 proteins.

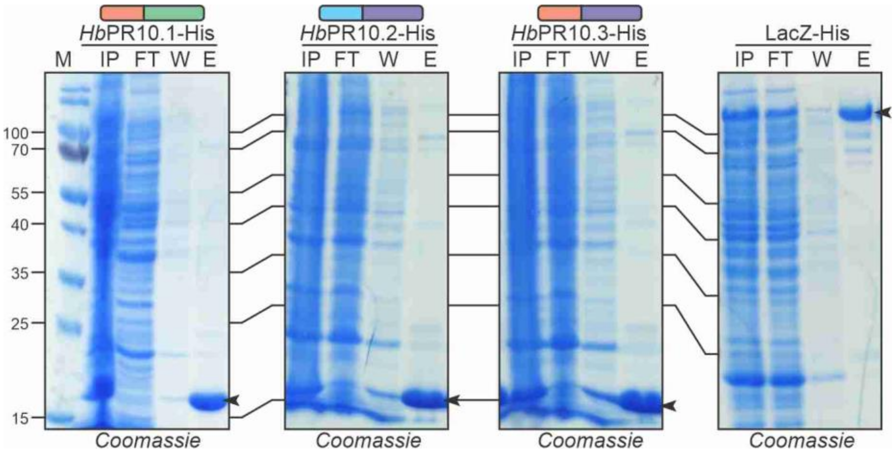

Supplemental Figure S2 Expression and purification of the three *Hb*PR10 proteins and the LacZ negative control.
